# Time Evolution of Initial Errors in Lorenz's 05 Chaotic Model

**DOI:** 10.1155/2015/729080

**Published:** 2015-08-04

**Authors:** Hynek Bednář, Aleš Raidl, Jiří Mikšovský

**Affiliations:** Department of Meteorology and Environment Protection, Faculty of Mathematics and Physics, Charles University in Prague, V Holešovičkách 2, 180 00 Prague, Czech Republic

## Abstract

Initial errors in weather prediction grow in time and, as they become larger, their growth slows down and then stops at an asymptotic value. Time of reaching this saturation point represents the limit of predictability. This paper studies the asymptotic values and time limits in a chaotic atmospheric model for five initial errors, using ensemble prediction method (model's data) as well as error approximation by quadratic and logarithmic hypothesis and their modifications. We show that modified hypotheses approximate the model's time limits better, but not without serious disadvantages. We demonstrate how hypotheses can be further improved to achieve better match of time limits with the model. We also show that quadratic hypothesis approximates the model's asymptotic value best and that, after improvement, it also approximates the model's time limits better for almost all initial errors and time lengths.

## 1. Introduction

Forecast errors in numerical weather prediction models (NWPMs) grow in time because of the inaccuracy of the initial state, chaotic nature of the weather system itself, and the model imperfections. Due to the nonlinear terms in the governing equations, the forecast error will saturate after some time. Time of saturation or* the limit of predictability of deterministic forecast* in NWPM is defined by [1] as time when the prediction state diverges as much from the verifying state as a randomly chosen but dynamically and statistically possible state. Forecasters also use other time limits to measure the error growth.* Forecast-error doubling time τ*_*d*_ is time when initial error doubles its size. *τ*_95%_, *τ*_71%_, *τ*_50%_, and *τ*_25%_ are times when the forecast error reaches 95%, 71%, 50%, and 25% of the limit of predictability. The time limit *τ*_71%_ is the time when the forecast error exceeds 1/√2 of the saturation or asymptotic value (AV) and, by [2], it corresponds to the level of climatic variability. Lorenz [3] calculated forecast error growth of NWPM by comparing the integrations of model, starting from slightly different initial states. Present-day calculations use the approach developed by Lorenz [4], where we can obtain two types of error growth. The first is called* lower bound* and is calculated as the root mean-square error (RMSE) between forecast data of increasing lead times and analysis data valid at the same time. The second is called* upper bound* and is calculated as the root mean-square (RMS) difference between pairs of forecasts, valid at the same time but with times differing by some fixed time interval. For example, if this interval is one day, the analysis for a given day is compared with one day forecast valid for the same day, and then this one day forecast is compared with two days forecast valid for the same day and so on. This second method compares only model equations and therefore it represents growth without model error. The innovation to upper bound, that is also used, is calculated as the RMS difference between forecast and control forecast with higher resolution of the model (*perfect model framework*).

*Quadratic hypothesis* (QH) was the first attempt that was made by Lorenz [3] to quantify the error growth. QH is based on the assumption that, if the principal nonlinear terms in the atmospheric equations are quadratic, then the nonlinear terms in the equations governing the field of errors are also quadratic. Dalcher and Kalney [5] added a model error to Lorenz's QH. A version that is used by recent researchers is the Simmons's et al. modification [6] of [5]. The Lorenz's QH is therefore suitable for upper bound of error growth and the Simons's et al. modification for lower bound. Trevisan et al. [7] came out with idea that logarithmic term is more valid than quadratic and linear term in the equations governing the field of errors, but this* logarithmic hypothesis* (LH) has never been used in NWPM computations.

*Ensemble prediction systems* (EPS) are used in order to estimate forecast uncertainties. They consist of a given number of deterministic forecasts where each individual forecast starts from slightly different initial states. EPS also includes a stochastic scheme designed to simulate the random model errors due to parameterized physical processes. Recent studies of predictability and forecast error growth (e.g., [8–11]) are mostly done by models of European Centre for Medium Range Weather Forecasts (ECMWF) and the Global Ensemble Forecast System (GEFS) from the National Centers for Environmental Prediction (NCEP). They include deterministic and ensemble forecast with 1 to 70 members. Operational model of ECMWF uses 50 members plus control forecast. More detailed study [10] uses 5 members plus control forecast. The initial conditions of ensemble members are defined by linear combination of the fastest singular vectors. Horizontal resolution with spectral truncation varies from T95 to T1279 and the number of vertical levels varies from 19 to 91 (analyses use higher resolution than forecasts). The output data are interpolated to 1° latitude × 1° longitude or 2.5° latitude × 2.5° longitude resolution separately for the Northern Hemisphere (20°, 90°) and Southern Hemisphere (−90°, −20°). Forecast is usually run for 90 days at winter (DJF) or summer (JJA) season with 0 (analysis) to 10, 15 (ECMWF), or 16 days (NCEP) of* forecast length* (FL) at 6 or 12 hours intervals. The most often used variable for analyzing the forecast error is geopotential height at 500 hPa level (Z500). Others are geopotential height at 1000 hPa level (Z1000) and the 850 hPa temperature (T850). To describe the forecast error growth over the calculated forecast length, the Simmons et al.'s modification [6] of Lorenz's QH [3] is used.

The questions that have arisen from studies of predictability and forecast error growth and that represent the key issues addressed in this work are: Is the LH [7] better approximation of initial error growth than QH [3]? Is there a possible modification of LH and QH that better approximates model data? If so, how much difference it creates in time limits that measure the forecast error growth? How precisely do the approximations describe forecast error growth over the FL (10, 15 or 16 days)? How do the approximations obtained from model values with various number of ensemble members differ from each other? Lorenz's chaotic atmospheric model (L05II) [12] will be used. For a more comprehensive introduction to the problem of weather predictability, we refer reader to the book by Palmer and Hagedorn [13]. After this introduction, Section 2 describes the model and experimental design, Section 3 describes ensemble prediction method, Section 4 introduces quadratic and logarithmic hypotheses, and Section 5 sets experimental designs.  Section 6 presents the results and their discussion and Section 7summarizes the conclusions.

## 2. Model

Because of the limitations of NWPMs and because we want to derive the impact of initial error (perfect model framework), we use modification [13] of low-dimensional atmospheric model (L96). L96 [14] is a nonlinear model, with *N* variables *X*_1_,…, *X*_*N*_ connected by governing equations:

(1) $$\begin{array}{c} \frac{dX_{n}}{dt}=-X_{n-2}X_{n-1}+X_{n+1}X_{n-1}-X_{n}+F. \end{array}$$

*X*_*n*__−2_, *X*_*n*−1_, *X*_*n*_, *X*_*n*+1_ are* unspecified (i.e., unrelated to actual physical variables) scalar meteorological quantities*, *F* is a constant representing external forcing, and *t* is time. The index is cyclic so that *X*_*n*−*N*_ = *X*_*n*+*N*_ = *X*_*n*_ and variables can be viewed as existing around a circle. Nonlinear terms of (1) simulate advection. Linear terms represent mechanical and thermal dissipation. The model quantitatively, to a certain extent, describes weather systems, but, unlike the well-known Lorenz's model of atmospheric convection [15], it cannot be derived from any atmospheric dynamic equations. The motivation was to formulate the simplest possible set of dissipative chaotically behaving differential equations that share some properties with the “real” atmosphere. NWPMs interpolate the output data mostly to 1° latitude × 1° longitude grid. In L96, it means *N* = 360. Such a high resolution would create large number of waves with similar maxima “pressure highs” and minima “pressure lows”; however, to share some properties with the “real” atmosphere, we would rather have 5 to 7 main highs and lows that correspond to planetary waves (Rossby waves) and a number of smaller waves that correspond to synoptic-scale waves. Therefore, we introduce spatial continuity modification (L05II) [12] of L96. Equation (1) is rewritten to the form:

(2) $$\begin{array}{c} \frac{dX_{n}}{dt}={\left[X,X\right]}_{L,n}-X_{n}+F, \end{array}$$

where

(3) $$\begin{array}{c} {\left[X,X\right]}_{L,n}={\overset{J}{\underset{j=-J}{\sum }}}^{\prime }{\overset{J}{\underset{i=-J}{\sum }}}^{\prime }\frac{\left(-X_{n-2L-i}X_{n-L-j}+X_{n-L+j-i}X_{n+L+j}\right)}{L^{2}}. \end{array}$$

**Figure 1:**
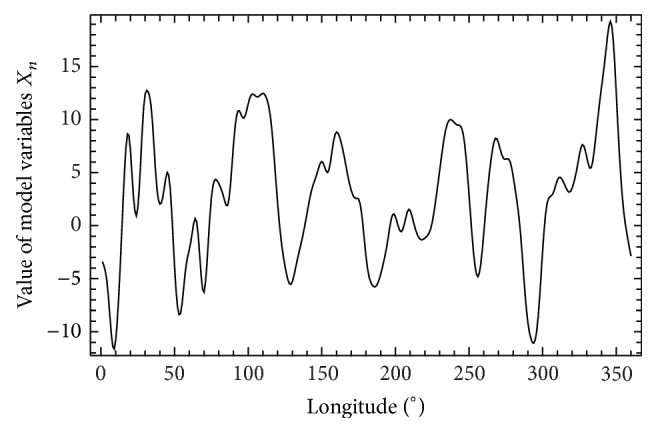
An example of longitudinal structure of model variable *X*.

If *L* is even, ∑′ denotes a modified summation, in which the first and last terms are to be divided by 2. If *L* is odd, ∑′ denotes an ordinary summation. Generally, *L* is much smaller than *N* and *J* = *L*/2 if *K* is even and *J* = (*L* − 1)/2 if *L* is odd. For our computation, we choose *N* = 360, so each sector covers 1° degrees of longitude. To keep a desirable number of main pressure highs and lows, Lorenz suggested keeping ratio *N*/*L* = 30 and therefore *L* = 12. Parameter *F* = 15 is selected as a compromise between too long doubling time (smaller *F*) and undesirable shorter waves (larger *F*). We first choose arbitrary values of the variables *X*, and, using a fourth order Runge-Kutta method with a time step Δ*t* = 0.05 or 6* hours*, we integrate forward for 14400* steps*, or 10* years*. We then use the final values, which should be free of transient effect.  Figure 1 shows values of model variables with selected parameters. For this setting and by the method of numerical calculation presented in [16], the global largest Lyapunov exponent is *λ*_max_ = 0.32. The definition of a chaotic system according to [3] states, that a bounded dynamical system with a positive Lyapunov exponent is chaotic. Because the value of the largest Lyapunov exponent is positive and the system under study is bounded, it is chaotic. Strictly speaking, we also need to exclude the asymptotically periodic behavior, but such a task is impossible to fulfill for the numerical simulation. The choice of parameters *F* and* time unit* = 5* days* is made to obtain similar value of the largest Lyapunov exponent as state of the art NWPMs.

## 3. Ensemble Prediction Method

The ensemble prediction method (EPM) employed is similar to [14] and is used to calculate average initial error growth. We make an initial “run” by choosing error *e*_*n*0_ and letting *X*_*n*0_′ = *X*_*n*0_ + *e*_*n*0_ be the “observed” initial value of *N* variables. We then integrate forward from the true and the observed initial state, for between 25 and 37.5 days (*K* = 100 to *K* = 150 steps). This time length covers initial error growth till the limit of predictability. We obtain *N* sequences *X*_*n*0_,…, *X*_*nK*_ and *X*_*n*0_′,…, *X*_*nK*_′, after which we let *e*_*nk*_ = *X*_*nk*_′ − *X*_*nk*_ for all values of *k* and *n*. In NWPM, forecast error growth is obtained from an average of values from 90 days and from various number of ensemble members. To simulate that, we make a total of *M*_1_ = 100, *M*_2_ = 250, and *M*_3_ = 500 runs in the above described manner. In each run, new values of *X*_*n*0_ are set as the old values of *X*_*nK*_. Finally, we let *e* ^2^(*τ*) = 1/*N*(*e*_1*k*_^2^ + ⋯+*e*_*Nk*_^2^) be the average of the *N* values, where *τ* = *k*Δ*t* is the predictable range and log*E* ^2^(*τ*) = 1/*M*(log*e* ^2^(*τ*)_1_ + ⋯+log*e* ^2^(*τ*)_*M*_) is the average of *M* values. Logarithmic average is chosen because of its suitability for comparison with growth governed by the largest Lyapunov exponent. For further information, see [17–19].

## 4. Error Growth Hypotheses

According to Lorenz [14], there is an eventual cessation of the exponential error growth due to processes represented by nonlinear terms in the weather governing equations. Most important are the quadratic terms, which represent the advection of the temperature and velocity fields. Under the assumption that the principal nonlinear terms in the atmospheric equations are quadratic, nonlinear terms in equations governing the field of errors are also quadratic. To describe this, Lorenz [14] defined QH

(4) $$\begin{array}{c} \frac{dE\left(t\right)}{dt}=aE\left(t\right)-bE{\left(t\right)}^{2}, \end{array}$$

where *E*(*t*) is a distance at time *t* between two originally nearby trajectories and *a* and *b* are constants. As an alternative, Trevisan et al. [7] introduced LH

(5) $$\begin{array}{c} \frac{dE\left(t\right)}{dt}=-cE\left(t\right)ln\left(gE\left(t\right)\right), \end{array}$$

where *c* and *g* are constants. The explanation follows, if we let $Q(t)=ln(\bar{E(t)})$ and $\overset{-}{E}$ is the normalized *E*, then *dQ*(*t*)/*dt* = *a*(1 − *e* ^*Q*(*t*)^) represents the QH. In [7], it is assumed that linear fit *dQ*(*t*)/*dt* = −*aQ*(*t*) is superior to the QH.

As modifications of QH (4), we use Simmons's et al. [6] version (QHM),

(6) $$\begin{array}{c} \frac{dE\left(t\right)}{dt}=a_{m}E\left(t\right)-b_{m}E{\left(t\right)}^{2}+s_{m}, \end{array}$$

that is used for approximation of growth with combination of initial and model error and where *a*_*m*_, *b*_*m*_ and *s*_*m*_ are constants. We also add the constant term to LH (5) (LHM)

(7) $$\begin{array}{c} \frac{dE\left(t\right)}{dt}=-c_{m}E\left(t\right)ln\left(g_{m}E\left(t\right)\right)+l_{m}, \end{array}$$

where *c*_*m*_, *g*_*m*_, *l*_*m*_ are constants. The reason for choosing (6) and (7) is based on assumption that, at *t* = 0, *dE*(*t*)/*dt* = 0 for ((6) and (7)) but *dE*(*t*)/*dt* ≠ 0 for model data. By adding the constant term, we may solve this difference.

In [7, 20, 21], it is shown on low-dimensional models that if the initial error is sufficiently small and therefore the early stages of error growth are exponential, QH is superior. If the initial error is not small enough, it is better to use LH. Generally, whether an error is small enough to guarantee the exponential growth depends on specific meteorological conditions and/or model under study.

## 5. Experimental Design

We want to achieve the conditions as similar to NWPM as possible. The size of initial error for NWPM (perfect model framework) is by [9] approximately between 2% and 0.01% of AV of the forecast error for control forecast and between 10% and 3% of AV for ensemble members. Different values of AV fraction are a result of varying resolution and because it is calculated for different variables (Z500, Z1000, and T850). In our case, the AV is *E*_asym_ = 8.4. This is calculated by four independent methods with the same results. The first method is numerical computation of ensemble prediction approach. Second and third methods are based on formula:

(8) $$\begin{array}{c} E_{asym}=\bar{{\left(f_{1}-X_{avr}\right)}^{2}}+\bar{{\left(f_{2}-X_{avr}\right)}^{2}}=2\bar{{\left(f_{1}-X_{avr}\right)}^{2}}, \end{array}$$

where *f*_1_ is “forecast” from *X*_*n*0_, *f*_2_ from *X*_*n*0_′, and *X*_avr_ is average value of *X*_*n*_. The bars above the (8) members mean the average value. The explanation for (8) can be found in [6, 10]. The fourth method is based on assumption [2] that variability of *X*_*n*_ is 71% of *E*_asym_.

Recalculation of initial errors to L05II model leads to the sizes of *X*_*n*_ between 0.001 and 0.84. For initial error sizes *e*_*n*0_, we therefore choose randomly from five normal distributions ND(*µ*; *σ*). ND_1_ = (0; 0.1), ND_2_ = (0; 0.2), ND_3_ = (0; 0.4), ND_4_ = (0; 0.6), ND_5_ = (0; 1), where *µ* is mean and *σ* is standard deviation. These choices of *e*_*n*0_ are made, because [20, 21] shows that change over QH and LH takes place between *e*_*n*0_ = 0.1 and *e*_*n*0_ = 1 for L96. NWPM routinely defines initial conditions of ensemble members by the fastest singular vectors. We do not adopt it, because, by [10, 22], it affects early stages of forecast error growth and, in our case, we want to have model data as close as possible to the tested hypotheses. From these initial conditions, the average initial error growth *E* is calculated from ensemble prediction method by the fourth order Runge-Kutta integration schema with a time step Δ*t* = 0.05 or 6* hours* for *M*_1_ = *M*_2_ = 100, *M*_3_ = *M*_4_ = 250, and *M*_5_ = 500. Because we want to study agreement of ((4)–(7)) with model data, we make differences of model data *y*_*k*_ = (*E*(*τ* + Δ*t*) − *E*(*τ*))/Δ*t* at points *x*_*k*_ = (*E*(*τ*) + *E*(*τ* + Δ*t*))/2, *k* = 1,…, *K* and *K* = 56 (*τ*_1_ = 14* days*), *K* = 76 (*τ*_2_ = 19* days*), *K* =* limit of predictability* (*τ*_3_), and we calculate parameters *a*, *b*, *c*, *g*, *a*_*m*_, *b*_*m*_, *s*_*m*_, *c*_*m*_, *g*_*m*_ and *l*_*m*_. The choice of the first two values of *K* is made, because we want to keep ratio *τ*_95%_/*forecast length* the same for NWPM and L05II.

The solutions of ((4)–(6)) are

(9) $$\begin{array}{c} E\left(t\right)=\frac{a}{\left(\left(a/e_{0}\right)-b\right)exp\left(-at\right)+b}, \end{array}$$

(10) $$\begin{array}{c} E\left(t\right)=\frac{{\left(ge_{0}\right)}^{exp\left(-ct\right)}}{g}, \end{array}$$

(11) Et=12bmam−−am2+4bmsm×tan12−am2+4bmsmt−arctan−am+2bme0−am2+4bmsmhhhhhh.−arctan−am+2bme0−am2+4bmsm.

LHM (7) cannot be solved analytically and therefore it is solved numerically by the fourth order Runge-Kutta integration schema with a time step Δ*t*_*m*_ = 0.25. We have five types of normal distribution for reaching sizes of initial error ND_1,…,5_, five settings for EPM *M*_1,…,5_, three FL *τ*_1,…,3_, and five ways of getting data of initial error growth: EPM, ((9)–(11)) and numerical solution of (NS (7)). To answer the key questions, we compute time limits for all combinations. We take *M*_1–5_,  *τ*_3_, EPM as the most reliable dataset in our experiment for all *e*_0_ and we calculate differences with other combinations at the same time limits.

## 6. Results and Discussion

**Figure 2:**
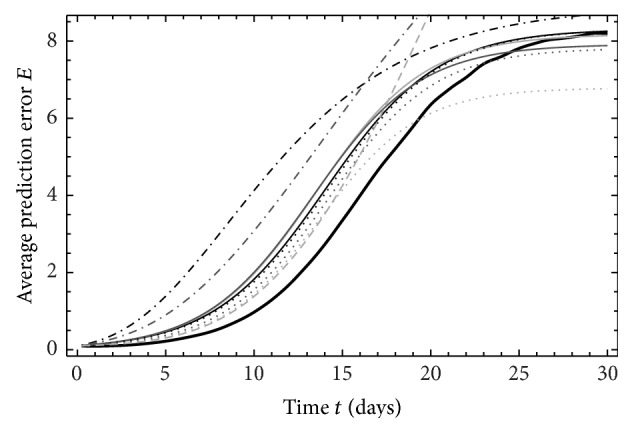
Time variations of the average prediction error *E* for ND_1_, *M*_5_. The thick line represents data from EPS, the thin lines from (9), the dotdashed lines from (10), the dotted lines from (11), and dashed lines from NS (7). The light grey lines represent the data extrapolate from time length *τ*_1_, the grey lines represent the data extrapolated from time length *τ*_2_ and black lines represent the data in time length *τ*_3_.

**Figure 3:**
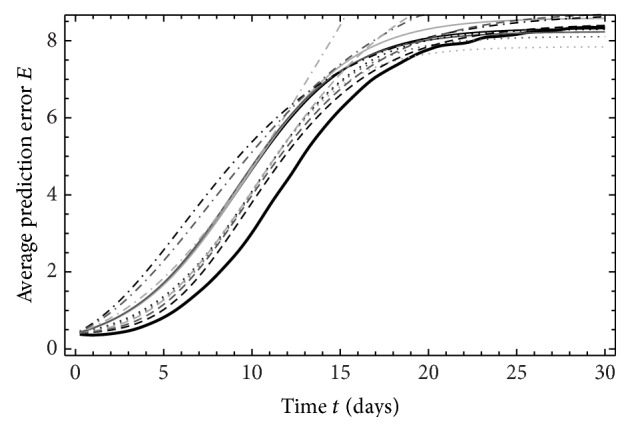
As Figure 2 for ND_3_, *M*_5_.

**Figure 4:**
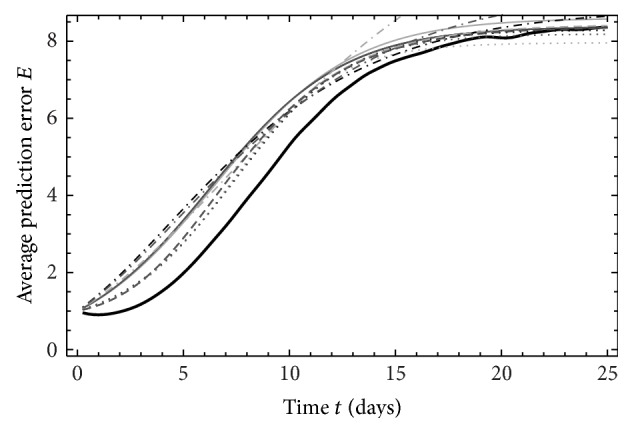
As Figure 2 for ND_5_, *M*_5_.

Tables 1 and 2 show with darker grey rows the resulting values ($\overset{-}{M}$, *τ*_3_, EPM) for all time limits and for all *e*_0_ represented by ND_1,…,5_. $\overset{-}{M}$ is average value of *M*_1,…,5_ and we use it, because the difference between *M*_1,…,5_ is of the order of 0.1 and *M*_3_ and *M*_4_ do not show closer values to *M*_5_ than *M*_1_ and *M*_2_. Lines in Tables 1 and 2 marked ($\overset{-}{M}$, EPM, *τ*_3_) − ($\overset{-}{M},\alpha ,\beta$) where *α* successively represents (9), (10), (11), and NS (7), and *β* = *τ*_1,…,3_ show the difference between most reliable data ($\overset{-}{M}$, *τ*_3_, EPM) and data from combinations of *α*, *β*. Columns marked by *ε*_*d*_, *ε*_25%_, *ε*_50%_, *ε*_71%_, and *ε*_95%_ display standard deviation of $\overset{-}{M}$. Last third of Table 2 shows average values $\bar{ND}$ of ND_1,…,5_ with standard deviations *σ*_*d*_, *σ*_25%_, *σ*_50%_, *σ*_71%_, and *σ*_95%_. From Tables 1 and 2, we can see that QH with solution (9) has an almost constant difference (EPM, *τ*_3_) − (*α*, *β*) equal on average $\bar{ND}$ to value between 2.3 and 2.5 days with *σ* between 0.1 and 0.3 days. The value of difference (EPM, *τ*_3_) − ((9), *τ*_1_) is higher only for *τ*_95%_. LH with solution (10) does not give good fit to model values and it is the worst choice if we compare it with other approximations. QHM with solution (11) has an almost constant difference (EPM, *τ*_3_) − (*α*, *β*) equal on average $\bar{ND}$ to value between 1.5 and 1.9 days with *σ* between 0 and 0.2 days. Different from these averages are values of difference (EPM, *τ*_3_) − ((11), *τ*_2_) at *τ*_95%_ and values of difference (EPM, *τ*_3_) − ((11), *τ*_1_) at *τ*_71%_. Values of difference (EPM, *τ*_3_) − ((11), *τ*_1_) at *τ*_95%_ are not displayed, because *E*_asym,*τ*1_ is smaller than model value at *τ*_95%_ (Figures 2, 3, and 4). The solution NS (7) of LHM does not exist for ND_1,2_ and *τ*_2,3_ (Figure 2). For others ND and *τ*, there is also an almost constant difference (EPM, *τ*_3_) − (*α*, *β*) equal on average $\bar{ND}$ to value between 1.3 and 1.5 days with *σ* between 0 and 0.2 days. Different from these averages are values of difference (EPM, *τ*_3_) − (NS (7), *τ*_2_) at *τ*_95%_ and values of difference (EPM, *τ*_3_) − (NS (7), *τ*_1_) at *τ*_71%_ and *τ*_95%_. Let us first take a look on constant differences and their source. Figures 2 to 4 display time variations of the average prediction error *E* for ND_1,3,5_. In these figures, we can see that, in contrast to approximations, the model values show negative growth rate for the first day, but turning into increase thereafter. At around two days, the model values reach the same value as it had initially. NWPMs also show this type of behavior [23] and approximation cannot capture that.

To summarize our findings, even though solutions (9) and NS (7) give better approximations to model data than (7) for all *τ*_1,…,3_, they have major disadvantages. Solution (9) underestimates the model data for *τ*_2_, *τ*_95%_, and *τ*_1_, *τ*_95%_ and therefore we cannot calculate the time when this approximation reaches 95% of *E*_asym_. Solution NS (7) does not exist for ND_1,2_ and *τ*_2,3_. If we subtract two days (time when the model values reach the same value that they had initially) from ($\overset{-}{M}$, EPM, *τ*_3_) − ($\overset{-}{M},\alpha ,\beta$), (7) would become superior, because we would get similar result as (9) and NS (7) but without the above mentioned disadvantages. One may argue that, because of subtraction of 2 days, we should recalculate the approximations. We did that and the results are close to the ones with subtraction. It is also good to mention that *τ*_1_ is always higher than *τ*_25%_ and lower than *τ*_95%_ and *τ*_2_ is always higher than *τ*_71%_. In the case of values at *τ*_2_, *τ*_95%_, *τ*_1_, *τ*_71%_ and *τ*_1_, *τ*_95%_, the highest difference from almost constant values is for *τ*_1_, *τ*_95%_ and the best results are given by QH with solution (9).

**Figure 5:**
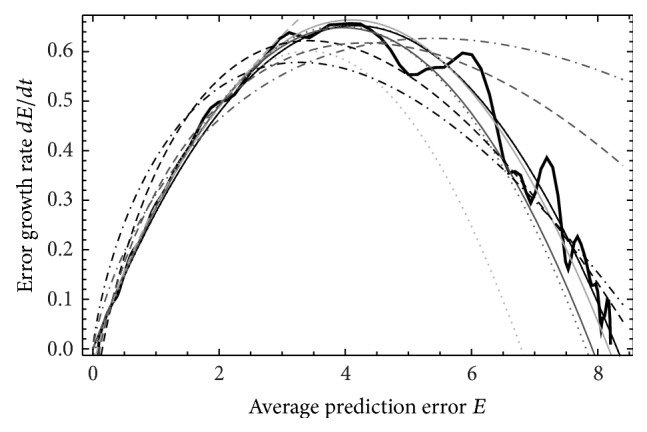
The error growth rate *dE*/*dt* versus *E* for ND_1_, *M*_5_. The thick line represents data from EPS, the thin lines from (9), the dotdashed lines from (10), the dotted lines from (11), and dashed lines from NS (7). The light grey lines represent the data extrapolated from time length *τ*_1_, the grey lines represent the data extrapolated from time length *τ*_2_, and black lines represent the data in time length *τ*_3_.

**Figure 6:**
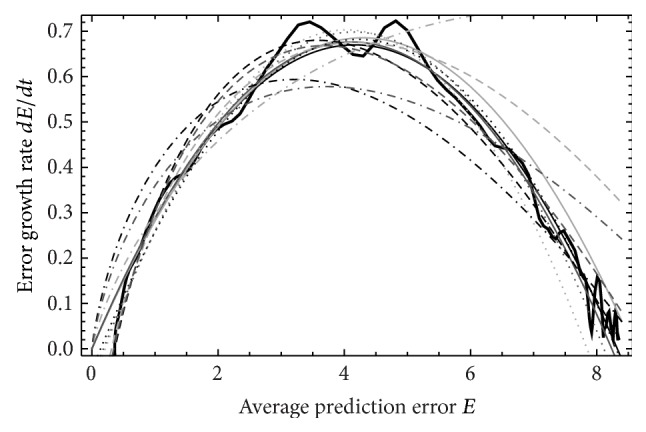
As Figure 5 for ND_3_, *M*_5_.

**Figure 7:**
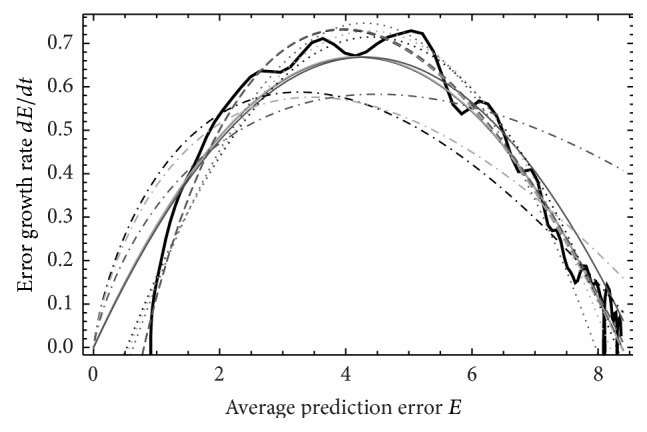
As Figure 5 for ND_5_, *M*_5_.

Table 3 focuses on the average values over *M*_1,…,5_ of *E*_asym,*τ*1,…,3_ for ND_1,…,5_ and average $\bar{ND}$. This value is for example used to find out if the variability of the model is equal to the variability of the atmosphere [10]. The differences *ε*_1,…,3_ from model values *E*_asym_ (Table 3, Figures 5, 6, and 7) indicate really poor approximation by (5) and (7) and usable approximations by (4) and (6), but with the already mentioned disadvantage of underestimations by (6). For (4), *ε*_3_ lies between −0.1 and 0 (relatively against *E*_asym_ it means between −1.2% and 0%), *ε*_2_ between −0.1 and 0 (between −1.2% and 0%), and *ε*_1_ between −0.4 and 0.4. (−4.8% and 4.8%).

## 7. Conclusion

This paper studies errors of estimations of time limits and asymptotic value of initial errors growth in chaotic atmospheric model L05II introduced by Lorenz [12] with the parameters as close to NWPM as possible. Five types of initial conditions are represented by five normal distributions. Five settings of EPM showed the differences of order 0.1 and therefore the average value was chosen as model data. Quadratic hypothesis shows the best agreement with model's asymptotic value *E*_asym_ and good agreement with model time limits. Approximation can be even improved by subtraction of constant value and after that the quadratic hypothesis is closest to model data from all hypotheses. Purpose and size of this constant are explained. Logarithmic hypothesis has the lowest agreement with the model data for time limits and asymptotic value. Modified quadratic hypothesis is good in approximating the model asymptotic value but it is not the best. For time limits, it is the best choice for approximation as long as we do not use the subtraction of the constant. Disadvantage is that, for some cases, this hypothesis underestimates model data and therefore some time limits are not available. Modified logarithmic hypothesis does not give good agreement with model's asymptotic value but gives similar agreement with model's time limit as modified quadratic hypothesis. Disadvantage is that, for the first two initial conditions, it is not solvable and therefore is usable only for larger initial errors. Quadratic hypothesis after subtraction of the constant value overestimates the model data for 0.5 days on average. Higher value is shown only for the shortest prediction time length *τ*_1_ and time limit *τ*_95%_. The size is 1.9 days on average. Relative difference between model's asymptotic value and asymptotic value calculated from quadratic hypothesis is between 0 and 1.2% for prediction time *τ*_3_ and *τ*_2_ and between −4.8% and 4.8% for prediction time *τ*_1_. So, only for the lastly mentioned prediction time, we should calculate with this difference.

## Figures and Tables

**Table 1:** Average values over *M*_1,…,5_ of time limits (in days) for model values (EPM), for normal distributions ND_1,…,3_, and for prediction time length *τ*_3_ (bold rows). Difference between this model values and average values over *M*_1,…,5_ received from (9)–(11) and NS (7) with parameters *a*, *b*, *c*, *g*, *a*_*m*_, *b*_*m*_, *s*_*m*_, *c*_*m*_, *g*_*m*_, and *l*_*m*_ calculated from approximations of (4)–(7) for *τ*_1_, *τ*_2_, *τ*_3_, and *M*_1,…,5_ (columns *τ*_*d*_, *τ*_25%_, *τ*_50%_, *τ*_71%_, *τ*_95%_). Standard deviation of $\overset{-}{M}$ (columns *ε*_*d*_, *ε*_25%_, *ε*_50%_, *ε*_71%_, *ε*_95%_).

	ND_1_									

(Days)	*τ*_*d*_	*ε*_*d*_	*τ*_25%_	*ε*_25%_	*τ*_50%_	*ε*_50%_	*τ*_71%_	*ε*_71%_	*τ*_95%_	*ε*_95%_

(**EPM, ** **τ**_3_)	**4.8**	**0.1**	**12.9**	**0.1**	**16.3**	**0.1**	**19.2**	**0.2**	**25.8**	**0.2**

(EPM, *τ*_3_) − ((9), *τ*_3_)	2.6	0	2.4	0.3	2.4	0.2	2.4	0.3	1.9	0.3
(EPM, *τ*_3_) − ((10), *τ*_3_)	3.8	0	6.4	0.3	6.2	0.2	5.6	0.3	5.1	0.9
(EPM, *τ*_3_) − ((11), *τ*_3_)	2.2	0.2	2	0.4	1.9	0.3	1.9	0.3	1.4	0.6
(EPM, *τ*_3_) − (NS (7), *τ*_3_)	—	—	—	—	—	—	—	—	—	—

(EPM, *τ*_3_) − ((9), *τ*_2_)	2.6	0	2.4	0.2	2.4	0.3	2.5	0.3	2.6	1.2
(EPM, *τ*_3_) − ((10), *τ*_2_)	3.3	0	4.5	0.2	4.3	0.2	4.5	0.3	8.1	0.2
(EPM, *τ*_3_) − ((11), *τ*_2_)	2.1	0.1	1.8	0.1	1.8	0.1	1.9	0.3	2.5	0.6
(EPM, *τ*_3_) − (NS (7), *τ*_2_)	—	—	—	—	—	—	—	—	—	—

(EPM, *τ*_3_) − ((9), *τ*_1_)	2.5	0.1	2.5	0.1	2.6	0.2	2.8	0.6	5.1	2.9
(EPM, *τ*_3_) − ((10), *τ*_1_)	—	—	—	—	—	—	—	—	—	—
(EPM, *τ*_3_) − ((11), *τ*_1_)	1.7	0.2	1.5	0.2	1.3	0.4	0.1	0.5	—	—
(EPM, *τ*_3_) − (NS (7), *τ*_1_)	1.3	0	1.1	0	1.3	0.1	2	0.3	6.6	0.4

	ND_2_									

(Days)	*τ*_*d*_	*ε*_*d*_	*τ*_25%_	*ε*_25%_	*τ*_50%_	*ε*_50%_	*τ*_71%_	*ε*_71%_	*τ*_95%_	*ε*_95%_

(**EPM, ** **τ**_3_)	**4.9**	**0.1**	**10.6**	**0.1**	**14**	**0.2**	**16.9**	**0.2**	**24.3**	**0.5**

(EPM, *τ*_3_) − ((9), *τ*_3_)	2.6	0	2.5	0.3	2.6	0.4	2.7	0.4	3	0.4
(EPM, *τ*_3_) − ((10), *τ*_3_)	3.6	0	4.6	0.1	5.1	1.1	4.5	0.1	4.4	1
(EPM, *τ*_3_) − ((11), *τ*_3_)	1.9	0.4	1.6	0.5	1.6	0.4	1.7	0.4	2.1	0.4
(EPM, *τ*_3_) − (NS (7), *τ*_3_)	—	—	—	—	—	—	—	—	—	—

(EPM, *τ*_3_) − ((9), *τ*_2_)	2.6	0	2.5	0.2	2.6	0.3	2.6	0.3	2.2	1.4
(EPM, *τ*_3_) − ((10), *τ*_2_)	3.5	0.1	4.3	0.2	4	0.3	3.8	0.3	7.2	0.4
(EPM, *τ*_3_) − ((11), *τ*_2_)	1.9	0.3	1.6	0.3	1.6	0.3	1.6	0.3	−0.4	2.5
(EPM, *τ*_3_) − (NS (7), *τ*_2_)	—	—	—	—	—	—	—	—	—	—

(EPM, *τ*_3_) − ((9), *τ*_1_)	2.5	0.1	2.4	0.3	2.5	0.3	2.7	0.3	4.1	3
(EPM, *τ*_3_) − ((10), *τ*_1_)	2.9	0.1	2.8	0.2	2.5	0.3	2.6	0.3	7	0.3
(EPM, *τ*_3_) − ((11), *τ*_1_)	1.8	0.1	1.5	0.1	1.6	0.3	1.3	0.5	—	—
(EPM, *τ*_3_) − (NS (7), *τ*_1_)	1.5	0.2	1.5	0.1	1.5	0.2	1.9	0.4	6.5	0.7

	ND_3_									

(Days)	*τ*_*d*_	*ε*_*d*_	*τ*_25%_	*ε*_25%_	*τ*_50%_	*ε*_50%_	*τ*_71%_	*ε*_71%_	*τ*_95%_	*ε*_95%_

(**EPM, ** **τ**_3_)	**4.9**	**0.1**	**8.2**	**0.2**	**11.6**	**0.2**	**14.4**	**0.2**	**21.6**	**0.4**

(EPM, *τ*_3_) − ((9), *τ*_3_)	2.6	0.1	2.3	0.1	2.4	0.1	2.1	0.1	2.7	0.2
(EPM, *τ*_3_) − ((10), *τ*_3_)	3.5	0.1	4	0.1	3.9	0	3.2	0.2	2.6	0.8
(EPM, *τ*_3_) − ((11), *τ*_3_)	2.1	0.1	1.6	0.1	1.6	0.2	1.6	0.2	2.1	0.3
(EPM, *τ*_3_) − (NS (7), *τ*_3_)	1	0.2	1.1	0.2	1.3	0.2	1	0.3	1	0.6

(EPM, *τ*_3_) − ((9), *τ*_2_)	2.6	0.1	2.4	0.1	2.3	0.1	2.2	0.2	2.4	0.9
(EPM, *τ*_3_) − ((10), *τ*_2_)	3.4	0	3.6	0.1	3.3	0.2	2.8	0.2	5	0.2
(EPM, *τ*_3_) − ((11), *τ*_2_)	2	0.1	1.5	0.2	1.6	0.2	1.6	0.2	1.2	1.4
(EPM, *τ*_3_) − (NS (7), *τ*_2_)	1.5	0.2	1.5	0.2	1.6	0.2	1.4	0.2	2.6	0.6

(EPM, *τ*_3_) − ((9), *τ*_1_)	2.7	0	2.3	0.1	2.3	0.1	2.2	0.2	3.4	0.6
(EPM, *τ*_3_) − ((10), *τ*_1_)	2.9	0	2.8	0.1	2.5	0.1	2.6	0.1	7	0.2
(EPM, *τ*_3_) − ((11), *τ*_1_)	1.8	0.1	1.4	0.2	1.6	0.2	1.4	0.3	—	—
(EPM, *τ*_3_) − (NS (7), *τ*_1_)	1.6	0.1	1.5	0.2	1.6	0.2	1.6	0.2	4.1	0.7

**Table 2:** Average values over *M*_1,…,5_ of time limits (in days) for model values (EPM), for normal distributions ND_4,5_, and for prediction time length *τ*_3_ (bold rows), difference between this model values and average values over *M*_1,…,5_ received from (9)–(11) and NS (7) with parameters *a*, *b*, *c*, *g*, *a*_*m*_, *b*_*m*_, *s*_*m*_, *c*_*m*_, *g*_*m*_, and *l*_*m*_ calculated from approximations of (4)–(7) for *τ*_1_, *τ*_2_, *τ*_3_ and *M*_1,…,5_ (columns *τ*_*d*_, *τ*_25%_, *τ*_50%_, *τ*_71%_, *τ*_95%_). Standard deviation of *M*_1,…,5_ (columns *ε*_*d*_, *ε*_25%_, *ε*_50%_, *ε*_71%_, *ε*_95%_) and average value $\bar{\text{ND}}$ and standard deviation (columns *σ*_*d*_, *σ*_25%_, *σ*_50%_, *σ*_71%_, *σ*_95%_) of ND_1,…,5_.

	ND_4_									

(Days)	*τ*_*d*_	*ε*_*d*_	*τ*_25%_	*ε*_25%_	*τ*_50%_	*ε*_50%_	*τ*_71%_	*ε*_71%_	*τ*_95%_	*ε*_95%_

(**EPM, ** **τ**_3_)	**4.9**	**0.1**	**6.8**	**0.1**	**10.2**	**0.2**	**12.9**	**0.1**	**19.7**	**0.3**

(EPM, *τ*_3_) − ((9), *τ*_3_)	2.4	0.1	2.3	0	2.3	0.1	2.3	0.1	2.4	0.3
(EPM, *τ*_3_) − ((10), *τ*_3_)	3.3	0.1	3.5	0.1	3.3	0.1	2.6	0.1	1.7	0.3
(EPM, *τ*_3_) − ((11), *τ*_3_)	1.8	0.1	1.5	0.1	1.4	0.1	1.5	0.2	1.8	0.3
(EPM, *τ*_3_) − (NS (7), *τ*_3_)	1.3	0.1	1.3	0.2	1.5	0.2	1.5	0.2	1.2	0.4

(EPM, *τ*_3_) − ((9), *τ*_2_)	2.5	0.1	2.4	0.1	2.3	0.1	2.3	0.1	2.1	0.6
(EPM, *τ*_3_) − ((10), *τ*_2_)	3.1	0	3.2	0.1	2.8	0.1	2.3	0.1	3.4	0.1
(EPM, *τ*_3_) − ((11), *τ*_2_)	1.6	0	1.3	0.1	1.4	0.2	1.5	0.2	0.4	0.8
(EPM, *τ*_3_) − (NS (7), *τ*_2_)	1.3	0.1	1.4	0.2	1.6	0.1	1.5	0.2	1.6	0.3

(EPM, *τ*_3_) − ((9), *τ*_1_)	2.5	0.1	2.2	0.1	2.2	0.1	2.2	0.2	3.6	0.6
(EPM, *τ*_3_) − ((10), *τ*_1_)	2.7	0.1	2.6	0.2	2.2	0.2	2.1	0.2	5.6	0.1
(EPM, *τ*_3_) − ((11), *τ*_1_)	1.6	0.1	1.5	0.2	1.6	0.2	1.6	0.2	—	—
(EPM, *τ*_3_) − (NS (7), *τ*_1_)	1.6	0.1	1.5	0.1	1.6	0.1	1.6	0.1	3.4	0.5

	ND_5_									

(Days)	*τ*_*d*_	*ε*_*d*_	*τ*_25%_	*ε*_25%_	*τ*_50%_	*ε*_50%_	*τ*_71%_	*ε*_71%_	*τ*_95%_	*ε*_95%_

(**EPM, ** **τ**_3_)	**5**	**0.1**	**5.2**	**0.2**	**8.2**	**0.2**	**10.9**	**0.3**	**18.2**	**0.3**

(EPM, *τ*_3_) − ((9), *τ*_3_)	2.5	0	2.4	0	2.1	0.2	2.5	0.9	2.8	0.4
(EPM, *τ*_3_) − ((10), *τ*_3_)	3	0	2.9	0	2.5	0.1	1.6	0.2	1.4	0.3
(EPM, *τ*_3_) − ((11), *τ*_3_)	1.7	0.1	1.6	0.1	1.2	0.1	1.3	0.2	1.3	0.4
(EPM, *τ*_3_) − (NS (7), *τ*_3_)	1.6	0.1	1.6	0.1	1.6	0.1	1.6	0.2	2	0.2

(EPM, *τ*_3_) − ((9), *τ*_2_)	2.4	0.1	2.3	0.1	2	0.2	1.9	0.2	3.6	0.2
(EPM, *τ*_3_) − ((10), *τ*_2_)	2.6	0.1	2.6	0.1	1.9	0.1	1.4	0.2	4.6	0.2
(EPM, *τ*_3_) − ((11), *τ*_2_)	1.5	0.1	1.4	0.1	1.3	0.1	1.5	0.2	—	0.3
(EPM, *τ*_3_) − (NS (7), *τ*_2_)	1.6	0.1	1.6	0.1	1.6	0.1	1.6	0.2	1.9	0.2

(EPM, *τ*_3_) − ((9), *τ*_1_)	2.4	0.1	2.5	0.1	2.1	0.1	2	0.2	2.6	0.6
(EPM, *τ*_3_) − ((10), *τ*_1_)	2.8	0.1	2.8	0.1	2.2	0.2	1.5	0.2	2.6	0.2
(EPM, *τ*_3_) − ((11), *τ*_1_)	1.6	0	1.5	0	1.3	0.1	1.4	0.2	1.4	4.3
(EPM, *τ*_3_) − (NS (7), *τ*_1_)	1.6	0.1	1.6	0.1	1.5	0.1	1.6	0.2	1.9	1.1

$\bar{\text{ND}}$										

(Days)	*τ*_*d*_	*σ*_*d*_	*τ*_25%_	*σ*_25%_	*τ*_50%_	*σ*_50%_	*τ*_71%_	*σ*_71%_	*τ*_95%_	*σ*_95%_

(EPM, *τ*_3_) − ((9), *τ*_3_)	2.5	0.1	2.4	0.1	2.3	0.1	2.4	0.2	2.5	0.3
(EPM, *τ*_3_) − ((10), *τ*_3_)	3.4	0.2	4.3	1	4.2	1.2	3.5	1.3	3	1.4
(EPM, *τ*_3_) − ((11), *τ*_3_)	1.9	0.2	1.6	0.1	1.5	0.2	1.6	0.2	1.9	0.3
(EPM, *τ*_3_) − (NS (7), *τ*_3_)	1.3	0.2	1.3	0.2	1.5	0.1	1.3	0.2	1.4	0.4

(EPM, *τ*_3_) − ((9), *τ*_2_)	2.5	0.1	2.4	0	2.3	0.1	2.3	0.2	2.4	0.2
(EPM, *τ*_3_) − ((10), *τ*_2_)	3.2	0.2	3.7	0.6	3.3	0.6	3	0.9	5.2	1.9
(EPM, *τ*_3_) − ((11), *τ*_2_)	1.8	0.2	1.5	0.1	1.5	0.2	1.6	0.1	1	0.8
(EPM, *τ*_3_) − (NS (7), *τ*_2_)	1.5	0.1	1.5	0.1	1.5	0.0	1.5	0.1	2	0.4

(EPM, *τ*_3_) − ((9), *τ*_1_)	2.5	0.1	2.3	0.1	2.3	0.2	2.3	0.3	3.9	0.5
(EPM, *τ*_3_) − ((10), *τ*_1_)	2.8	0.1	2.7	0.1	2.3	0.3	2.4	0.7	6.5	1.5
(EPM, *τ*_3_) − ((11), *τ*_1_)	1.6	0.1	1.5	0	1.5	0.1	1.2	0.4	—	—
(EPM, *τ*_3_) − (NS (7), *τ*_1_)	1.5	0.1	1.4	0.1	1.5	0.1	1.7	0.2	4.5	1.6

**Table 3:** Average values over *M*_1,…,5_ of *E*_asym,*τ*1,…,3_, calculated from approximations (4)–(7) for all initial conditions ND_1,…,5_ and for all prediction time lengths *τ*_1,…,3_ (bold columns), average value $\bar{\text{ND}}$ over ND_1,…,5_ for all prediction time lengths *τ*_1,…,3_ and difference *ε*_1,…,3_ of *E*_asym,*τ*1,…,3_ − *E*_asym_.

	*E*_asym,*τ*3_	*ε*_3_	*E*_asym,*τ*2_	ε_2_	*E*_asym,*τ*1_	ε_1_
(ND_1_, (4))	**8.3 ± 0.1**	−0.1 ± 0.1	**8.4 ± 0.3**	0.0 ± 0.3	**8.1 ± 0.5**	−0.3 ± 0.5
(ND_1_, (5))	**9.1 ± 0.4**	0.7 ± 0.4	**17.0 ± 1.4**	8.6 ± 1.4	**—**	—
(ND_1_, (6))	**8.3 ± 0.1**	−0.1 ± 0.1	**8.4 ± 0.9**	0.0 ± 0.9	**8.0 ± 2.1**	−0.4 ± 2.1
(ND_1_, (7))	**8.8 ± 0.2**	0.4 ± 0.2	**12.7 ± 2.4**	4.3 ± 2.4	**26.8 ± 7.7**	18.4 ± 7.7

(ND_2_, (4))	**8.3 ± 0.0**	−0.1 ± 0.0	**8.3 ± 0.2**	−0.1 ± 0.2	**8.8 ± 0.7**	0.4 ± 0.7
(ND_2_, (5))	**8.9 ± 0**	0.5 ± 0.0	**10.9 ± 1.0**	2.5 ± 1.0	**63.4 ± 21.0**	55.0 ± 21.0
(ND_2_, (6))	**8.3 ± 0.3**	−0.1 ± 0.3	**8.2 ± 0.2**	−0.2 ± 0.2	**7.8 ± 0.7**	−0.6 ± 0.7
(ND_2_, (7))	**8.6 ± 0.3**	0.2 ± 0.3	**10.2 ± 1.0**	1.8 ± 1.0	**15.9 ± 2.9**	7.5 ± 2.9

(ND_3_, (4))	**8.4 ± 0.0**	0.0 ± 0.0	**8.3 ± 0.1**	−0.1 ± 0.1	**8.3 ± 0.2**	−0.1 ± 0.2
(ND_3_, (5))	**8.8 ± 0.2**	0.4 ± 0.2	**10.2 ± 0.2**	1.8 ± 0.2	**18.2 ± 1.7**	9.8 ± 1.7
(ND_3_, (6))	**8.3 ± 0.0**	−0.1 ± 0.0	**8.2 ± 0.2**	−0.2 ± 0.2	**7.7 ± 0.2**	−0.7 ± 0.2
(ND_3_, (7))	**8.5 ± 0.1**	0.1 ± 0.1	**8.9 ± 0.2**	0.5 ± 0.2	**9.8 ± 0.3**	1.4 ± 0.3

(ND_4_, (4))	**8.4 ± 0.1**	0.0 ± 0.1	**8.3 ± 0.1**	−0.1 ± 0.1	**8.7 ± 0.3**	0.3 ± 0.3
(ND_4_, (5))	**8.8 ± 0.1**	0.4 ± 0.1	**9.6 ± 0.2**	1.2 ± 0.2	**15.3 ± 1.6**	6.9 ± 1.6
(ND_4_, (6))	**8.3 ± 0.1**	−0.1 ± 0.1	**8.2 ± 0.1**	−0.2 ± 0.1	**8.1 ± 0.2**	−0.3 ± 0.2
(ND_4_, (7))	**8.4 ± 0.1**	0.0 ± 0.1	**8.5 ± 0.1**	0.1 ± 0.1	**9.3 ± 0.4**	0.9 ± 0.4

(ND_5_, (4))	**8.3 ± 0.0**	−0.1 ± 0.0	**8.3 ± 0.1**	−0.1 ± 0.1	**8.5 ± 0.1**	0.1 ± 0.1
(ND_5_, (5))	**8.7 ± 0.1**	0.3 ± 0.1	**9.3 ± 0.1**	0.9 ± 0.1	**11.7 ± 0.5**	3.3 ± 0.5
(ND_5_, (6))	**8.3 ± 0.0**	−0.1 ± 0.0	**8.2 ± 0.1**	−0.2 ± 0.1	**7.9 ± 0.1**	−0.5 ± 0.1
(ND_5_, (7))	**8.4 ± 0.0**	0.0 ± 0.0	**8.3 ± 0.1**	−0.1 ± 0.1	**8.4 ± 0.1**	0.0 ± 0.1

($\bar{\text{ND}}$, (4))	**8.3 ± 0.0**	−0.1 ± 0.0	**8.3 ± 0.1**	−0.1 ± 0.0	**8.5 ± 0.2**	0.1 ± 0.2
($\bar{\text{ND}}$, (5))	**8.9 ± 0.1**	0.5 ± 0.1	**11.4 ± 2.2**	3.0 ± 2.2	**27.2 ± 18.1**	18.8 ± 18.1
($\bar{\text{ND}}$, (6))	**8.3 ± 0.0**	−0.1 ± 0.0	**8.2 ± 0.1**	−0.2 ± 0.1	**7.9 ± 0.1**	−0.5 ± 0.1
($\bar{\text{ND}}$, (7))	**8.5 ± 0.1**	0.1 ± 0.1	**9.7 ± 1.4**	1.3 ± 1.4	**14.0 ± 5.9**	5.6 ± 5.9
